# The prognostic impact of GSTM1/GSTP1 genetic variants in bladder Cancer

**DOI:** 10.1186/s12885-019-6244-6

**Published:** 2019-10-23

**Authors:** Nada Albarakati, Dareen Khayyat, Asharf Dallol, Jaudah Al-Maghrabi, Taoufik Nedjadi

**Affiliations:** 1 King Abdullah International Medical Research Center, King Saud bin Abdulaziz University for Health Sciences, Ministry of the National Guard - Health Affairs, Jeddah, Kingdom of Saudi Arabia; 2 King Fahd Medical Research Center, King Abdulaziz University, Jeddah, Saudi Arabia; 3 Centre of Excellence in Genomic Medicine Research and Medical Laboratory Technology Department, Faculty of Applied Medical Sciences, King Abdulaziz University, Jeddah, Saudi Arabia; 4 Department of Pathology, King Abdulaziz University, Jeddah, Saudi Arabia; 5 King Faisal Specialist Hospital & Research Center, Jeddah, Saudi Arabia

**Keywords:** Bladder cancer, GSTM1, GSTP1, HER2, Polymorphism, Prognosis

## Abstract

**Background:**

The glutathione S-transferases (GSTs) are a superfamily of phase II detoxifying enzymes that inactivates a wide variety of potential carcinogens through glutathione conjugation. Polymorphic changes in the *GST* genes have been reported to be associated with increased susceptibility to cancer development and anticancer drug resistance. In this study, we investigated the association between genetic variants in *GSTM1* and *GSTP1* and patients’ clinicopathological parameters. The prognostic values of such associations were evaluated among bladder cancer patients.

**Methods:**

Genotyping of *GSTM1* and *GSTP1* in bladder cancer patients was assessed using polymerase chain reaction followed by DNA sequencing. Overall survival was estimated using the Kaplan-Meier method and multiple logistic regression and correlation analysis were performed.

**Results:**

The *GSTM1* null genotype was significantly associated with poor overall survival compared with the wild-type *GSTM1* genotype. There was a trend towards better overall survival in patients with wild-type *GSTP1* allele (AA) compared with *GSTP1* (AG/GG) genotype. Interestingly, Kaplan-meier survival curve for *GSTM1* null patients adjusted for sub-cohort with amplified *HER2* gene showed poor survival compared with the *GSTM1* null/ non-amplified *HER2* gene. Also the same population when adjusted with HER2 protein expression, data showed poor survival for patients harboring *GSTM1* null/high HER2 protein expression compared with low protein expression.

**Conclusion:**

This study focuses on the impact of *GSTM1* null genotype on bladder cancer patients’ outcome. Further investigations are required to delineate the underlying mechanisms of combined *GSTM*^*−/−*^ and HER2 status in bladder cancer.

## Background

Bladder cancer is the 9th most common cancer and a leading cause of cancer-related death worldwide. It has been estimated that around 550,000 new bladder cancer cases and 199,922 deaths occurred in the year 2018 worldwide and these numbers are expected to double in the upcoming years [1]. The disease is highly recurring and do frequently progress to a muscle invasive phenotype which necessitate a vigilant and continuous monitoring protocol [2]. Despite advances in diagnostic and treatment modalities, bladder cancer remains source of co-morbidity and continues to pose challenges for clinicians given that patients’ outcome being solely dependent on the grading and staging system [3]. Therefore, a deeper understanding of the bladder cancer pathogenesis and associated mechanisms will undoubtedly improve patients’ outcome via prevention of disease progression and recurrence.

It is well documented that occupational exposure to chemical carcinogens including aromatic amines and polycyclic aromatic hydrocarbons is associated with the risk of bladder cancer development [2, 4]. Kellen et al. reported an increased risk of developing bladder cancer associated with cumulative exposure to aromatic amines, but not to PAHs and diesel [5]. In an independent study, Ferrís et al. concluded that bladder cancer is a result of the interaction between constitutional and environmental risk factors including aromatic amines and polycyclic aromatic hydrocarbons [6]. The involvement of environmental factors such as cigarette smoking in bladder carcinogenesis has been extensively investigated [7, 8]. Recent evidence supports the dynamic interplay between environmental factors and other co-factors, including genetic predisposition, in the pathogenesis of bladder cancer [9].

Protecting against carcinogen-induced and chemotherapy-induced oxidative stress involves a series of event characterized by the activation of phase-II cellular detoxifying enzymes; Glutathione S-transferases (GSTs) or N-acetyltransferases (NATs) [10]. GSTs enzymes superfamily consist of at least 16 genes located on more than 7 chromosomes [11]. Although they are structurally different with distinct evolutionary origins, all GSTs isoenzymes are functionally similar in protection against electrophiles and oxidative stressors. The cytosolic sub-family of GST is found to be active in a homo- or heterodimeric state and is sub-divided into eight classes designated as follow: GST alpha (α), mu (μ), kappa (κ), omega (ω), pi (π), sigma (σ), theta (θ), and zeta (ζ) [12]. GSTs play a critical protective anticancer role through glutathione conjugation with a range of potentially cytotoxic exogenous or endogenous molecules making them less toxic. Allelic polymorphisms in these genes elicit changes in enzyme activities leading to biotransformation and play important role in the development and progression of different cancers, such as lung, colorectal, leukemia, breast and bladder cancers. Furthermore, Sau et al. showed the contribution of GSTs overexpression in resistance against several anti-cancer drugs [13].

*GSTM1* gene is located on chromosome 1p13.3 and the most common polymorphic variant of *GSTM1* gene is the homozygous deletion (*GSTM1* null genotype) characterized by abolished enzyme activity [14]. Many studies have investigated the relationship between the genetic polymorphism of *GSTM1* and the risk of cancer, but the association remains controversial among different populations. Previous epidemiological studies showed an association between the homozygous deletion of *GSTM1* and increased risk of lung, colorectal and head and neck cancers [15–17]. However other studies failed to establish the association between *GSTM1* null and the risk of several types of cancers [18–21].

GSTP1 is encoded by a single gene located on chromosome 11 [22]. The common functional *GSTP1* polymorphism at codon 105 is an A to G substitution resulting in an amino acid switch from isoleucine to valine (Ile_105_Val) and lowering the catalytic activity of GSTP1enzyme [23]. The decreased detoxification capacity of the GSTP1 enzyme resulted in differences in chemotherapeutic responses. The increased expression of the *GSTP1* Val105 genotype was shown to be associated with a variety of tumors, such as ovarian, breast, colon, lymphoma, and pancreas [24]. The hypothesis that *GSTP1* variants modulate the risk of urinary bladder cancer has also been investigated [24, 25]. However, inconclusive results have been reported on the association between *GSTP1* gene polymorphisms and the risk of bladder cancer: while a number of studies identified an obvious association between *GSTP1* polymorphisms Ile_105_Val and bladder carcinoma risk [26–28], other studies illustrated that there are no association between *GSTP1* Ile_105_Val polymorphism and bladder cancer [29, 30].

HER2 is a trans-membrane glycoprotein receptor tyrosine kinase of the epidermal growth factor receptor family EGFR/ErbB. It plays an important role in the development and progression of many tumor types including breast, gastric and bladder cancers [31]. Recent sequencing efforts to uncover the complex genomic landscape of bladder cancer identified six distinct molecular subtypes. HER2-like is one of the main subtypes characterise by higher *ERBB2* amplification and signalling [32]. HER2 is considered one of the most important prognostic biomarkers that play an important role in the patho-physiology of bladder cancers and a potential therapeutic target in bladder cancer [31, 33, 34]. Also, interactions between GST gene family and other genes including *HER2* may be involved in cancer susceptibility and clinical management of cancer patients. In the present study, we aim to investigate the prognostic value of *GSTM1* and *GSTP1* genetic polymorphisms in patients with bladder cancer and evaluate their association with patients’ clinicopathological parameters. We also attempted to evaluate the clinical significance of HER2 status in cases confirmed to have GSTM1/ GSTP1 variants with bladder cancer prognosis.

## Methods

### Patients and sample collection

Formalin-fixed paraffin-embedded (FFPE) tissue samples were obtained from histologically confirmed bladder cancer patients who underwent bladder resection between 2005 and 2012 at King Abdulaziz University Hospital (KAUH), Jeddah, Saudi Arabia. The study group consists of 93 patients; only specimens containing more than 80% cellular composition were used in the analysis. All patients have not been subjected to any chemotherapy or radiotherapy prior to sample collection. Clinical and pathological data including age, gender, tumor grade, tumor stage, lymph node, vascular invasion, metastasis, and survival were gathered from patients’ medical records and summarized in Table 1. This study was ethically approved by the institutional research ethics committee, faculty of medicine, King Abdulaziz University (ref. N. 149–14).

**Table 1:** The clinicopathological characteristics of 93 patients with bladder cancer

The clinicopathological characteristics		N	%
Group age (Years)	≤60	37	39.78%
	> 60	55	59.14%
	Unknown	1	1.08%
Gender	Male	77	82.80%
	Female	16	17.20%
Tumor Grade	High Grade	56	60.22%
	Low Grade	29	31.18%
	Unknown	8	8.60%
Cancer type	MIBC	52	55.91%
	NMIBC	28	30.11%
	Unknown	13	13.98%
Subtypes	Transitional	74	79.57%
	Squamous	3	3.23%
	Transitional/ Squamous	15	16.13%
	Unknown	1	1.08%
Tumor Shape	Papillary	63	67.74%
	Non-papillary	3	3.23%
	Unknown	27	29.03%
Lymph Node	Positive	21	22.58%
	Negative	68	73.12%
	Unknown	4	4.30%
Vascular Invasion	Positive	18	19.35%
	Negative	70	75.27%
	Unknown	5	5.38%
Metastasis	Positive	21	22.58%
	Negative	67	72.04%
	Unknown	5	5.38%
Smoking	No	11	11.83%
	Yes	16	17.20%
	Unknown	66	70.97%
Family history of cancer	No	24	25.81%
	Yes	4	4.30%
	Unknown	65	69.89%
Survival	Alive	65	69.89%
	Deceased	28	30.11%

Abbreviation: *MIBC* Muscle Invasive Bladder Cancer, *NMIBC* Non-Muscle Invasive Bladder Cancer

### DNA isolation

Genomic DNA was extracted from FFPE tissue samples using QIAamp DNA FFPE Tissue Kit (Qiagen) according to the manufacturer’s instructions. Purified DNA was eluted in 50 μl elution buffer and stored at − 80 °C until use. Purity and concentration of eluted DNA was analyzed using a spectrophotometer system (Nanodrop 2000, Thermo Scientific, USA).

### GSTM1 and GSTP1 SNP genotyping

Genotyping for the detection of *GSTM1* (present/null) and *GSTP1* Ile_105_Val polymorphisms was performed as described previously [35]. Genotyping was carried out using real time PCR Kit (Qiagen) as per the manufacturer’s recommendation. Briefly 200 ng DNA was amplified in an overall volume of 25 μl/ reaction. *GSTM1* and *GSTP1* oligonucleotide primers were purchased from MWG-Biotech (Ebersberg, Germany) to amplify the *GSTM1* fragments, (Forward: 5′-CTGCCCTACTTGATTGATGGG-3′; Reverse: 5′-CTGGATTGTAGCAGATCATGC-3′), *GSTP1* (Forward: 5′-ACCCCAGGGCTCTATGGGAA-3′, Reverse: 5′-TGAGGGCACAAGAAGCCCCT-3′) PCR was performed on a Thermal Cycler 480 apparatus (Applied Biosystems, USA). Thermo cycler parameters included: an initial denaturation at 94 °C/ 15 min; followed by 35 cycles of denaturation at 94 °C/ 1 min, annealing at 57 °C /1 min, and extension at 74 °C/ 1 min; and a final extension at 72 °C/10 min. Confirmation of PCR products were examined by 2% agarose gel electrophoresis and visualized using a Syngene UV transilluminator.

### DNA sequencing

**Fig. 1:**
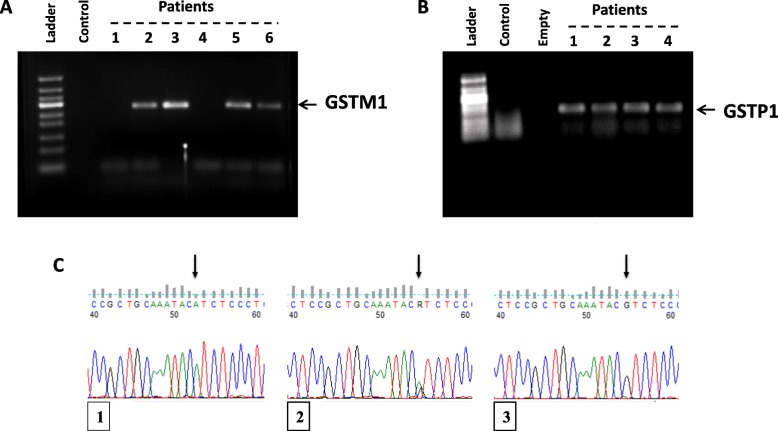
Representative screening for *GSTM1* and *GSTP1* Polymerase chain reaction products. Agarose gel of the PCR products for detection of GSTM1 deletion polymorphism [**a**] *GSTM1* verified by PCR analysis. **b** Agarose gel of the PCR products for detection of GSTP1 polymorphisms. **c** *GSTP1* validation by sequencing: (1) The wild allele homozygote AA, (2) heterozygote AG and (3) variant allele homozygote GG genotypes

To sequence the amplified *GSTP1* PCR products, sequencing kit (BigDye® Terminator v3.1 kit, Thermo Scientific, USA) was used according to the manufacturer’s instructions using Genetic analyzer 3500 (Applied Biosystems, UK). The resulting sequence data was analyzed using Applied Biosystems sequence analysis software (v 5.4). *GSTP1* genotypes were determined as wild type Ile/Ile (AA), heterozygous type Ile/Val (AG) or homozygous variant type Val/Val (GG) as shown in Fig. 1c. As for *GSTM1,* the PCR products were separated on a 2% agarose gel and determined as null/ present genotypes.

### Immunohistochemistry

HER2 immunostaining was undertaken earlier [33]. The expression of HER2 protein is mainly membranous, the protein expression in our bladder samples was evaluated as follows: No expression = negative Vs. Expression = weak, + 1; moderate, + 2; strong, + 3.

### Statistical analyses

Statistical data analysis was performed using SPSS (SPSS, version 25, USA). Appropriate, Chi-square test and Fisher’s exact test were used to establish any significant differences in polymorphism incidences between bladder cancer cases. Multivariate Cox regression model were used to evaluate the prognostic significance of GSTs genes, HER2 and other clinicopathological factors. Cumulative survival probabilities were estimated using the Kaplan-Meier method, with log-rank comparison test. Multiple logistic regression analysis was performed to assess the association between *GST* polymorphisms with aggressiveness of bladder cancer. Odds Ratios (OR) and their 95% Confidence Intervals (95% CI) were used to calculate the results. The wild type of all genotypes was used as the reference group. Interactions between *GSTM1* and *GSTP1* polymorphisms and aggressiveness bladder cancer phenotypes were analyzed using Spearman correlation analysis. In all tests, the values *p ≤ 0.05* were considered as statistically significant.

## Results

### Characteristics of the study population

In the current study, 93 patients with urinary bladder carcinoma were genotyped for two polymorphisms in two important genes of the glutathione-s-transferase family involved in xenobiotic metabolism. The distribution of the clinicopathological characteristics of the bladder cancer patients is presented in Table 1. Patients age ranges from 34 to 93 years with median age of 64 ± 12, the median follow-up time of 10.10 months (ranging 0–139 months) and preponderance of male over female in the ratio 5:1.

### Genotype distributions of the GSTM1and GSTP1 polymorphisms in patients

Polymerase chain reaction-based and Sanger gene sequencing-base assays were undertaken to assess the contribution of genetic polymorphism in *GSTM1* and *GSTP1* to the susceptibility of bladder cancer (Fig. 1). Lack of amplification products for the *GSTM1* gene was considered as a homozygous null genotype (−/−). Our data revealed that a total of 44 bladder cancer patients out of 93 (47.31%) had a *GSTM1*-deleted genotype (−/−). *GSTM1* specific bands showing on agarose gel electrophoresis was seen in 45 out of 93 patients (48.38%). No further investigations were carried out to discriminate between heterozygous deletion (+/−) and wild-type (+/+) *GSTM1* variants hence both heterozygous deletion and wild-type variants are considered *GSTM1* present (Fig. 1a).

As for the *GSTP1* frequencies, amplified PCR products containing GSTP1 were visualized on agarose gels (Fig. 1b) and the resultant DNA fragments were subjected to Sanger sequencing using BigDye terminator v3.1 (Life technologies). The GSTP1 wild allele homozygote (AA), heterozygote (AG) and variant allele homozygote (GG) genotypes were 36/93 (38.70%), 36/93 (38.70%) and 6/93 (6.45%) respectively (Fig. 1c). Merging both AG/GG genetic variants represent 45.16% (42/93) of the total analyzed cases, Table 2.

**Table 2:** The distribution (count and percentage) of *GSTM1* and *GSTP1* genotypes in the patients with bladder cancer

		N	%
GSTM1	Present	45	(48.38)
	Null	44	(47.31)
GSTP1	AA	36	(38.70)
	GG	6	(6.45)
	AG	36	(38.70)
	AG/GG	42	(45.16)

**Fig. 2:**
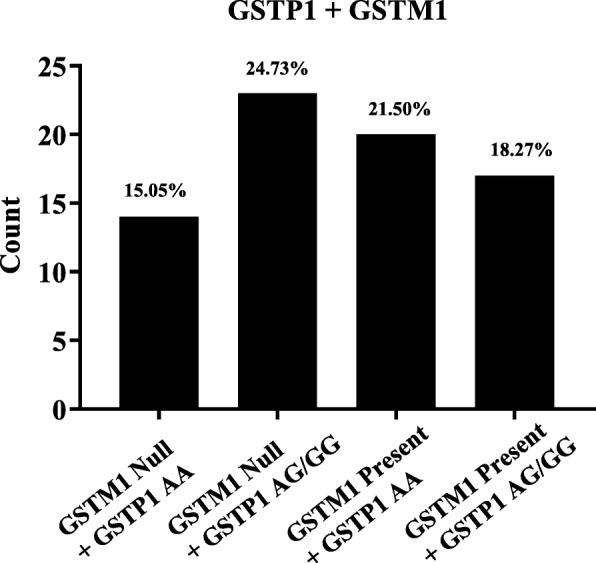
Distribution of the *GSTM1* + *GSTP1* variants in bladder cancer patients. The distribution of patients carrying *GSTM1* null and *GSTP1* recessive homozygote/ heterozygote AG/GG was 23 of 93 (24.73%). whereas the lowest was *GSTM1* null and the *GSTP1* wild allele 14 (15.05%)

A higher frequency within our cohort was found between those carrying *GSTM1* null and *GSTP1* recessive homozygote / heterozygote AG/GG 23 (24.73%), whereas the lower percentage was with *GSTM1* null and the *GSTP1* wild allele 14 (15.05%) shown in Fig. 2. No statistical significant was found between *GSTs* different groups.

### Effect of GSTM1and GSTP1 polymorphisms on patients’ survival

**Fig. 3:**
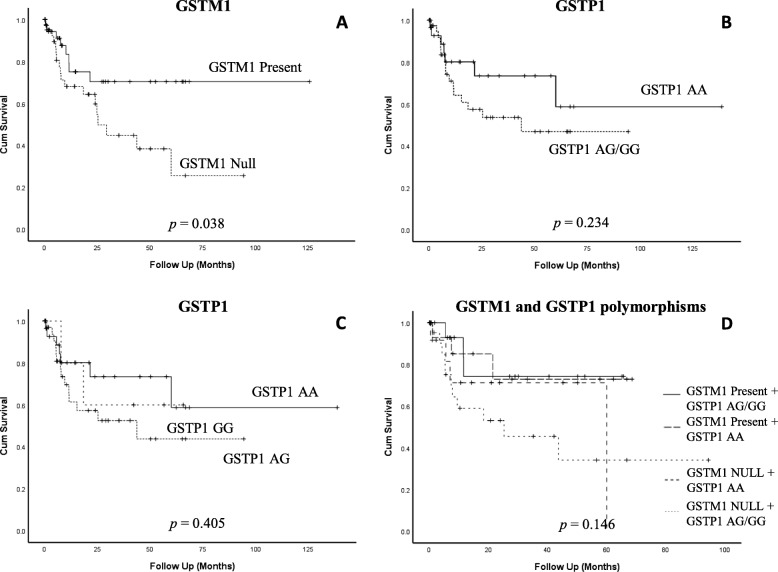
Kaplan-Meier survival curves demonstrating the overall survival of: **a** *GSTM1* null and present genotypes were evaluated in bladder cancer patients. **b** *GSTP1* genotypes, AA, AG and GG. **c** *GSTP1* AA and combined AG/GG. **d** Merging *GSTM1* and *GSTP1* overall survival. All *P* values tested by log-rank test. Patients alive at the last follow-up or lost to follow-up were censored in the survival comparison analysis

Kaplan-Meier curve showed that *GSTM1* null genotype was associated with poor overall survival in comparison to *GSTM1* present genotype, log rank *p* = 0.038 (Fig. 3a). As for *GSTP1*, though it is not statistically significant, patients harboring the wild type allele *GSTP1* AA have tendency for better survival in comparison to patients with *GSTP1* AG/GG genotype (Log rank, *p* = 0.234). *GSTP1* AG carriers had the worst overall survival compared to *GSTP1* AA or GG genotypes carriers (Fig. 3b, c. However, the associations were not statistically significant (log-rank test; *p* = 0.40). When merging *GSTM1* survival and *GSTP1* polymorphisms (Fig. 3d), there was trend towards poorer survival for patients with combined *GSTM1* null and *GSTP1* AG/GG (Log rank, *p* = 0.146).

### Relationships between GST genotypes, HER2 status and survival outcomes

Published data, including our own, revealed that bladder cancer exhibit high ratios of the Human Epidermal growth factor Receptor 2 (*HER2)* gene amplification, after breast and gastric cancers, and also demonstrates frequent overexpression of HER2 protein [33, 34]. Recently published data revealed that bladder cancer possess the highest frequency mutation in HER2 gene across 38 types of tumors analyzed [31]. Furthermore, HER2 is considered among the prognostic factors, along with staging and grading system, in urothelial bladder cancer [36]. In the current study we sought to investigate the relationship between *GSTM1* and *GSTP1* polymorphisms in respect to HER2 status of the same cohort. HER2 protein expression and gene amplification data [33] were available for 89 patients out of our 93 bladder cancer patients. Histograms showed the frequency of expression patterns of HER2 protein receptors in our cohort (Additional file 1: Figure S1). To establish the relationship between *GST* genotypes and HER2 status, bright field double in situ hybridization (BDISH) and immunohistochemistry (IHC) data were used to analyze *HER2* gene amplification and protein expression within the *GSTP1*/ *GSTM1* analyzed cohort. Our data indicated no association between HER2 protein level and both *GSTP1* (*p* = 0.07) and *GSTM1* (*p* = 0.75) polymorphic status (Table 3). However, *HER2* gene amplification was significantly associated with the *GSTP1* AA, AG & GG variants (*p* = 0.03). Such a relationship was not established for amplified *HER2* gene and *GSTM1* null/present variants (Table 3).

**Table 3:** Interaction between GSTM1 and GSTP1 polymorphisms and HER2 status

	GSTM1	GSTP1 (AA, AG & GG)	GSTP1 (AA & AG/GG)
	*P* value	*P* value	*P value*
HER2 Gene	0.42	0.03	0.08
HER2 Protein	0.75	0.11	0.07

**Fig. 4:**
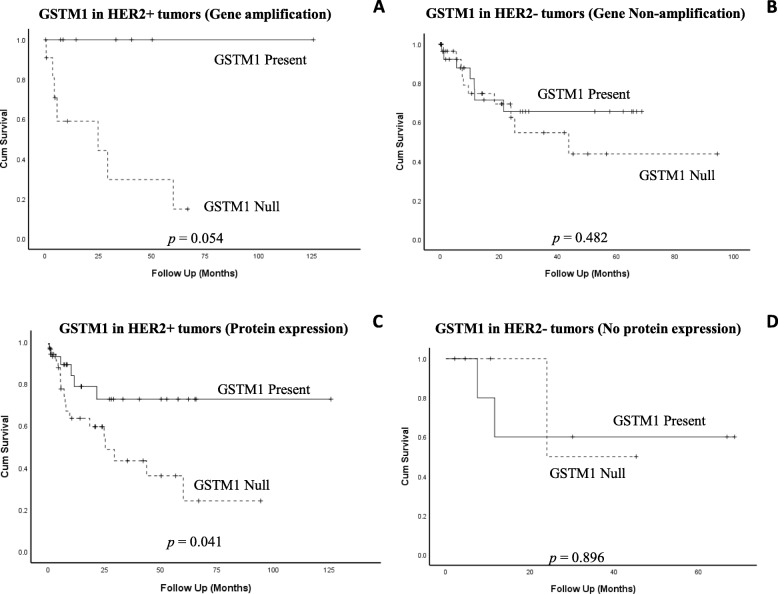
Kaplan-Meier survival curves demonstrating the overall survival of *GSTM1* adjusted with HER2 status. **a** *GSTM1* genotypes with *HER2* gene amplification. **b** *GSTM1* genotypes with *HER2* gene Non-amplification. **c** *GSTM1* genotypes with HER2 Protein expression. **d** *GSTM1* genotypes with No HER2 Protein expression

Interestingly, Kaplan-Meier survival curve for *GSTM1* status adjusted *to HER2* gene status (amplified or non-amplified) showed a significant impact on patients’ overall survival. Figure 4a, illustrates that poor overall survival was associated with combining *GSTM1* null and amplified *HER2* gene (Log rank, *p = 0.05*), though this was not the case with non-amplified *HER2* patients (Fig. 4b). To further confirm the observed relationship between amplified *HER2* gene and *GSTM1* null, we sought to analyze the relationship between HER2 protein level and *GSTM1* genotype. Similarly, survival curve (Fig. 4c) showed poor survival for patients carrying *GSTM1* null variant with high HER2 protein expression (Log rank, *p = 0.041*) compared to *GSTM1* null/ low HER2 protein expression counterpart (Fig. 4D). This synergistic effect of combined *GSTM1* genotype and increased *HER2 status* indicated a possible interaction between the two genes in bladder carcinogenesis. On the other hand, no difference in overall survival was observed in patients harboring combined *GSTP1* polymorphism and altered HER2 gene/protein levels (Additional file 2: Fig. S2A - 2D). The study cohort was then stratified into two groups based on the type of tumour (MIBC and NMIBC) and statistical analysis was performed to *to* determine which variables were *independently* associated with the patients’ outcome. In a multivariate analysis polymorphic GSTs gene expression has no independent prognostic value on bladder cancer overall survival. Similarly, No independent prognostic value of HER2 status was observed on overall survival (Table 4). Considering the small number of patients in each group (MIBC = 52, NMIBC = 28), it is meaningful to further explore its prognostic value in a large population size.

**Table 4:** Multivariate analyses compared with patients’ clinicopathological parameters, GSTs and HER2 status for bladder cancer overall survival

Variable	NMIBC				MIBC			
	Hazard ratio	95% Confidence Interval			Hazard ratio	95% Confidence Interval		
		Lower bound	Upper bound	*P* value		Lower bound	Upper bound	*P* value
Group age (≤60/> 60 Years)	0.81	− 0.144	0.564	0.215	1.64	− 0.035	0.480	0.087
Gender (F/M)	2.77	−0.519	0.206	0.356	1.45	−0.137	0.398	0.319
Tumor grade (High/Low)	0.46	−0.558	0.167	0.255	0.52	−0.433	0.113	0.232
Tumor subtypes (Transitional/Squamous)	1.75	−0.383	0.363	0.953	1.41	−0.173	0.367	0.459
Tumor shape (nonpapillary/papillary)	1.48	−0.099	0.594	0.142	1.07	−0.207	0.335	0.626
Lymph node (present/absent)	1.88	−0.655	0.015	0.059	0.98	−0.581	−0.093	0.009
Vascular invasion (present/absent)	–	–	–	–	0.66	−0.496	0.026	0.075
Metastasis (present/absent)	0.36	−0.530	0.205	0.345	1.72	−0.496	0.033	0.083
Smoking (yes/no)	1.41	−0.914	0.839	0.788	0.94	−0.638	0.284	0.380
Family history of cancer (yes/no)	–	–	–	–	0.38	−0.578	0.338	0.546
GSTM1 status (present/null)	1.21	−0.400	0.374	0.940	0.95	−0.510	0.002	0.052
GSTP1 status (AA/AG + GG)	0.55	−0.422	0.350	0.837	0.71	−0.035	0.549	0.081
HER2 gene status (non-amplified/amplified)	2.38	−0.457	0.297	0.642	0.54	−0.214	0.345	0.627
HER2 protein status (no expression/expression)	2.14	−0.459	0.310	0.669	1.04	−0.151	0.390	0.366

### GSTM1 and GSTP1 polymorphisms and clinicopathological parameters

Multiple logistic regression analysis was performed to assess the association between *GSTs* polymorphisms with patients’ clinical characteristics including tumor grade/ stage, muscle invasion, lymph node invasion, vascular invasion and metastasis. No association was observed between *GSTM1* polymorphism and patients’ clinicopathological characteristics. Similarly, no correlation was reported between *GSTP1* gene variants and patients’ clinicopathological features (Table 5).

**Table 5:** Association between GSTM1 and GSTP1 polymorphisms and clinicopathological features

		GSTM1					GSTP1				
		Null		Present			AA		AG/GG		
		N	%	N	%	*P* value	N	%	N	%	*P* value
Group age (Years)	≤60	17	18.2%	20	21.5%	0.51	16	17.2%	16	17.2%	0.49
	> 60	27	29.0%	24	25.8%		19	20.4%	26	27.9%	
Gender	Male	37	39.7%	37	39.7%	0.81	32	34.4%	33	35.4%	0.22
	Female	7	7.5%	8	8.6%		4	4.3%	9	9.6%	
Race	Asian	35	37.6%	40	43.0%	0.32	31	33.3%	38	40.8%	0.54
	African	8	8.6%	5	5.3%		5	5.3%	4	4.3%	
Tumor Grade	High Grade	29	31.1%	26	27.9%	0.18	23	24.7%	26	27.9%	0.67
	Low Grade	10	10.7%	17	18.2%		12	12.9%	11	11.8%	
Cancer Type	MIBC	28	30.1%	23	24.7%	0.17	18	19.3%	21	22.5%	1.00
	NMIBC	10	10.7%	16	17.2%		12	12.9%	14	15.0%	
Subtypes	Transitional	32	34.4%	39	41.9%	0.08	30	32.2%	35	37.6%	0.23
	Squamous	3	3.2%	0	0.0%		0	0.0%	3	3.2%	
	Transitional/ Squamous	9	9.6%	5	5.3%		5	5.3%	4	4.3%	
Lymph Node	Positive	13	13.9%	7	7.5%	0.14	8	8.6%	10	10.7%	0.93
	Negative	30	32.2%	35	37.6%		26	27.9%	31	33.3%	
Vascular Invasion	Positive	10	10.7%	7	7.5%	0.41	6	6.4%	9	9.6%	0.60
	Negative	32	34.4%	35	37.6%		28	30.1%	31	33.3%	
Metastasis	Positive	11	11.8%	9	9.6%	0.60	7	7.5%	13	13.9%	0.27
	Negative	31	33.3%	33	35.4%		27	29.0%	28	30.1%	
Survival	Alive	26	27.9%	37	39.7%	0.01^*^	29	31.1%	26	27.9%	0.072
	Deceased	18	19.3%	8	8.6%		7	7.5%	16	17.2%	

## Discussion

Globally, bladder cancer is a leading cause of mortality [37, 38]. It has long been perceived that bladder cancer is a result of occupational and environmental exposure to carcinogens and tobacco smoking, however, the exact mechanisms of bladder carcinogenesis remain unclear. Recent findings suggested that genetic factors contribute potentially, through mutations in key genes, in the etiology and pathogenesis of bladder cancer [7, 8, 39]. Glutathione S-Transferases (*GSTs*) are members of a large gene family of cytosolic phase II xenobiotic metabolizing enzymes involved in catalyzing and detoxifying a variety of carcinogens including reactive electrophilic compounds [11]. Members of the GST family play an important role in cellular defense through conjugation of xenobiotics with sulfhydryl group and promoting their excretion at later stage [11, 40]. It has been proposed that polymorphisms in members of *GST* of carcinogen-detoxifying gene family as well as in *NAT2* confer increased risk of bladder cancer [39]. Moreover, *increased expression of GST family members, especially GSTP1* and GSTM1*,* was reported in several human solid tumors and is believed to confer resistance to various platinum-base chemotherapy drugs and metformin through regulation of many genes and molecular pathways [41, 42]. Mechanistically, it is believed that polymorphisms in genes involved in drug-metabolizing enzymes may result in drastic changes in carcinogens biotransformation leading to increased cancer susceptibility [2].

In our investigation we examined the frequency of *GSTP1 and GSTM1 variants* in a cohort of 93 bladder cancer patient from Saudi Arabia. We also evaluated the association between *GSTP1* and *GSTM1* gene polymorphisms with a set of clinical and pathological parameters as well as the prognostic value of both genes polymorphisms in bladder cancer patients.

The frequency and distribution of *GSTM1* and *GSTP1* gene variants was represented in Table 2. In our study, the ratio of *GSTM1* present and null is equally distributed in our cohort 48.38 and 47.31% respectively. This data is in agreement with previous report on the frequency of the *GSTM1* null genotype in the Caucasian population [43]. In an independent study, Kang et al, revealed that the frequency of the *GSTM1* null genotype was 59.1% in patients with muscle invasive bladder cancer (MIBC) [44]. Nonetheless, it is well documented that the prevalence of *GSTM1* null genotype varies significantly among populations from different ethnic groups [45]. As for *GSTP1* gene polymorphism when we considered patients holding at least one copy of the dominant allele, data indicated that the frequency of AA and AG genotypes were found to be significantly high in our study group with a combined ratio of 77.4% for both genotypes compared to the GG genotype (6.45%). The reported frequency of *GSTP1* AA/AG genotypes is around 67% of the Iranian patients [26] and Indian patients [46]. However, a slight high frequency, approximately 80%, of *GSTP1* AA/AG variants was observed in in the Caucasian population with bladder cancer [47].

We next sought to evaluate the association between polymorphism of the *GSTP1* and *GSTM1* genes and patients’ outcome. Our results indicated a significant association between the null *GSTM1* genotype and poor overall survival among bladder cancer patients. The association between *GSTs* and poor survival was previously highlighted in many cancer types including bladder cancer [48–50]. As for *GSTP1* genotypes, our data show trend for better survival for patients with the wild allele homozygote AA in comparison to heterozygote AG and variant allele homozygote GG genotypes or to GG/AG combined though data are not significant. When *GSTP1* GG/AG and *GSTM1* null genotypes were present together, poor overall survival increased in comparison to *GSTP1* alone.

The accumulating data suggested that genetic polymorphism of *GSTs* leads to reduced detoxification potential which may result in increased DNA adduct levels in the target tissues and eventual mutations in the driver genes leading carcinogenesis. Therefore, the association of *GSTP1*/ *GSTM1* variants with highly malignant disease and poor prognosis in cancer patients was suggested [50].

Previous studies on patients from different ethnic origins revealed that individuals with the null *GSTM1* were at high risk of developing bladder cancer [26, 51–54]. This association was also seen between *GSTM1* null and other cancers such as breast [50], lung [55] and colorectal cancers [35]. Anwar et al. showed significantly higher *GSTM1* null distribution in bladder cancer patients than in healthy individuals [51]. The distribution of the null *GSTM1* in our cohort did not show any significant difference in comparison to the wild-type allele which may indicate that the null genotype is not the only factor in determining the increased risk and aggressiveness of bladder cancer but is certainly one of many combined genetic factors that contribute to the pathogenesis of the disease. To-Figueras et al. suggested a relation between *GSTM1* null genotype and *p53* mutation in increasing the risk of lung cancer susceptibility among smokers [55]. In an early observation by Ryk et al. the investigators demonstrated that the carriers of the variant allele of the *GSTP1* Ile_105_Val polymorphism were characterized by frequent mutations in the tumor suppressor gene *p53* and high-grade/ high stage tumors in bladder cancer [56]. In an independent investigation we performed high throughput mutational analysis of 50 oncogenes and tumor suppressor genes using cancer hotspot panel (CHP, v.2). Our data indicated that high proportion (~ 82%) of our bladder cancer cohort harbor *p53* mutation (data not published) which may suggest the involvement of *p53* mutation in association with GSTP1 in the risk of bladder cancer development and drug resistance. This suggestion is valid knowing that *GSTP1* gene contains a functional canonical p53 binding motif and the capacity of p53 to transcriptionally activate the human *GSTP1* gene [57].

In the same context and for the first time we investigated the relationship between different *GSTP1*/*GSTM1* variants and Human Epidermal growth factor Receptor 2 (*HER2*) gene/ protein status in bladder cancer patients. Our data indicated that patients with high HER2 protein expression/ gene amplification and null *GSTM1* genotype had significant poor survival compared to patients with low HER2 expression and null *GSTM1* genotype, suggesting that combining HER2 status with *GSTM1* genotype may have a prognostic value for bladder cancer patients. The exact mechanism of the influence of GSTM1 and HER2 on bladder cancer is yet to be elucidated. Together, our data showed that *GSTM1* gene deletion either alone or in combination with HER2 may serve as markers for bladder cancer prognosis.

We observed no association between the *GSTP1* Ile_105_Val genotype, *GSTM1* genotype alone or in combination with HER2 status and patients’ clinicopathological features. This is consistent with previous published reports [29, 58], and disagree with Safarinejad et al [26] who found a significant increase in tumor grade and stage of bladder cancer patients carrying *GSTP1* Val/Val genotype and *GSTM1*/*GSTT1* double null genotypes.

## Conclusions

The present study revealed that *GSTM1* null genotype is significantly associated with poor overall survival in urinary bladder cancer patients. Furthermore, combined *GSTM1* deletion and amplified *HER2* gene might be considered as the worse prognostic genotype combination in bladder cancer. To the best of our knowledge, this is the first study to investigate the association between *GSTs* genes polymorphisms and HER2 status in Saudi bladder cancer patients. One of the limitations of the current investigation is scarcity of the sample size and clinical data used for correlation analysis. Therefore, further analyses using larger sample size are needed to investigate the functional significance of combined GSTM1 deletion and HER2 on bladder cancer prognosis. Furthermore, larger epidemiological studies are needed to assess the relationship between these genes and response to therapies (chemotherapy and anti-HER2 therapy) which may support their use as potential predictive biomarkers for bladder cancer treatment.

## Supplementary information

**Additional file 1: Figure S1.** Histograms showed the frequency of expression patterns of HER2 protein receptors in 93 of bladder cancer by IHC.

**Additional file 2: Figure S2.** Kaplan-Meier survival curves demonstrating the overall survival of *GSTP1* adjusted with HER2 status. **(A)** *GSTP1* genotypes with *HER2* gene amplification. **(B)** *GSTP1* genotypes with *HER2* gene Non-amplification. **(C)** *GSTP1* genotypes with HER2 Protein expression. **(D)** *GSTP1* genotypes with No HER2 Protein expression.

## Data Availability

The datasets used and/or analyzed during the current study are available from the corresponding author on reasonable request.

## Abbreviations

BDISH: Bright field double in situ hybridization
GSTM1: Glutathione S-Transferase mu (μ)
GSTP1: Glutathione S-Transferase pi (π)
GSTs: Glutathione S-Transferases
HER2: Human epidermal growth factor receptor-2
IHC: Immunohistochemistry
TNM: Tumor, node and metastasis
