# Supplementary figures and images for: The prognostic impact of GSTM1/GSTP1 genetic variants in bladder Cancer
Source: BMC Cancer. 2019 Oct 23;19:991. doi: 10.1186/s12885-019-6244-6 (PMC6813104; doi:10.1186/s12885-019-6244-6)

## Slide 1
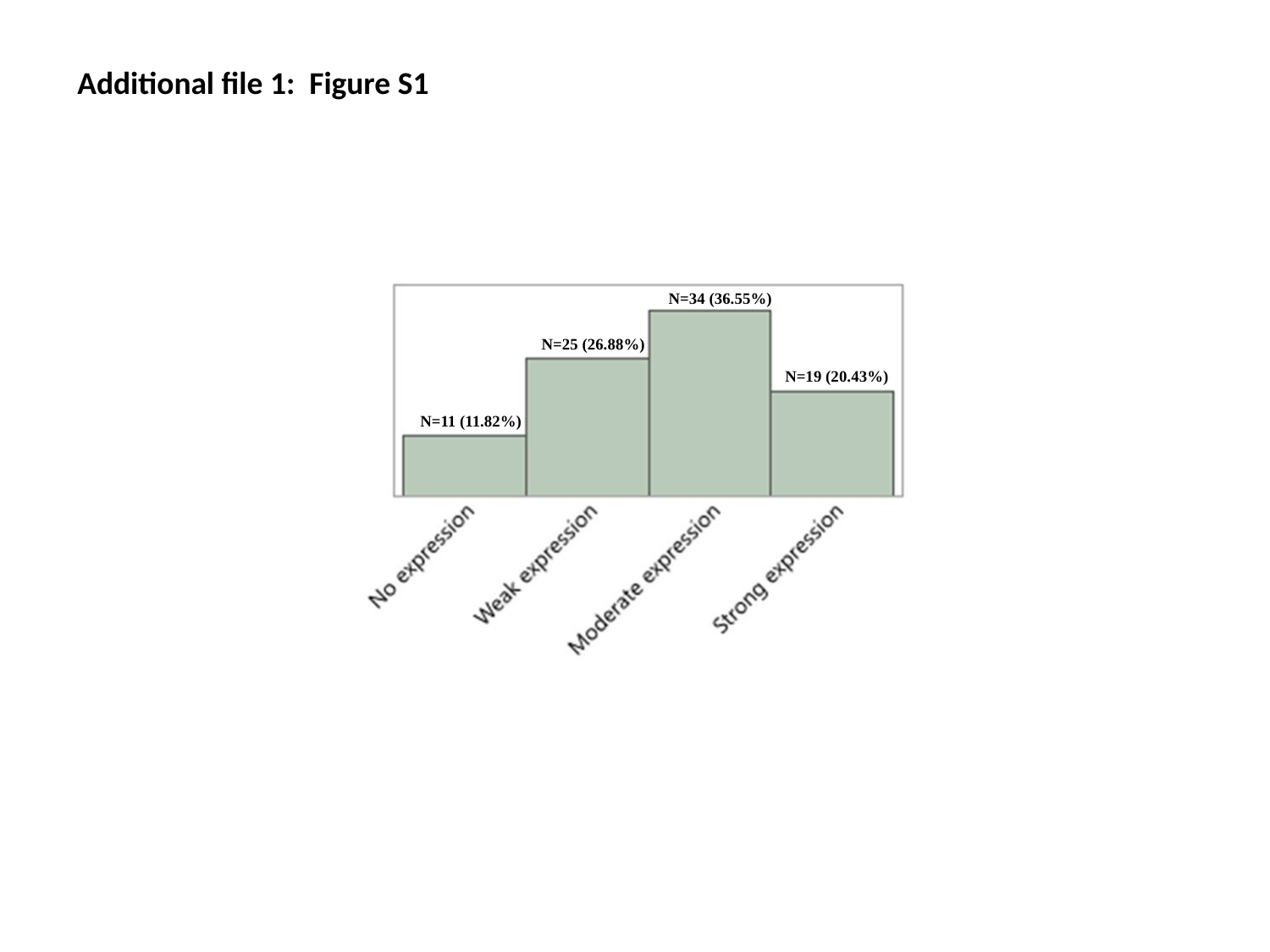

Additional file 1: Figure S1
N=34 (36.55%)
N=25 (26.88%)
N=19 (20.43%)
N=11 (11.82%)

Supplement: Supplementary file 1 — Additional file 1: Figure S1. Histograms showed the frequency of expression patterns of HER2 protein receptors in 93 of bladder cancer by IHC. [file 12885_2019_6244_MOESM1_ESM.pptx]
