# Supplementary material for: The prognostic impact of GSTM1/GSTP1 genetic variants in bladder Cancer
Source: BMC Cancer. 2019 Oct 23;19:991. doi: 10.1186/s12885-019-6244-6 (PMC6813104; doi:10.1186/s12885-019-6244-6)
Supplement: Supplementary file 2 — Additional file 2: Figure S2. Kaplan-Meier survival curves demonstrating the overall survival of GSTP1 adjusted with HER2 status. (A) GSTP1 genotypes with HER2 gene amplification. (B) GSTP1 genotypes with HER2 gene Non-amplification. (C) GSTP1 genotypes with HER2 Protein expression. (D) GSTP1 genotypes with No HER2 Protein expression. [file 12885_2019_6244_MOESM2_ESM.pptx]

## Slide 1
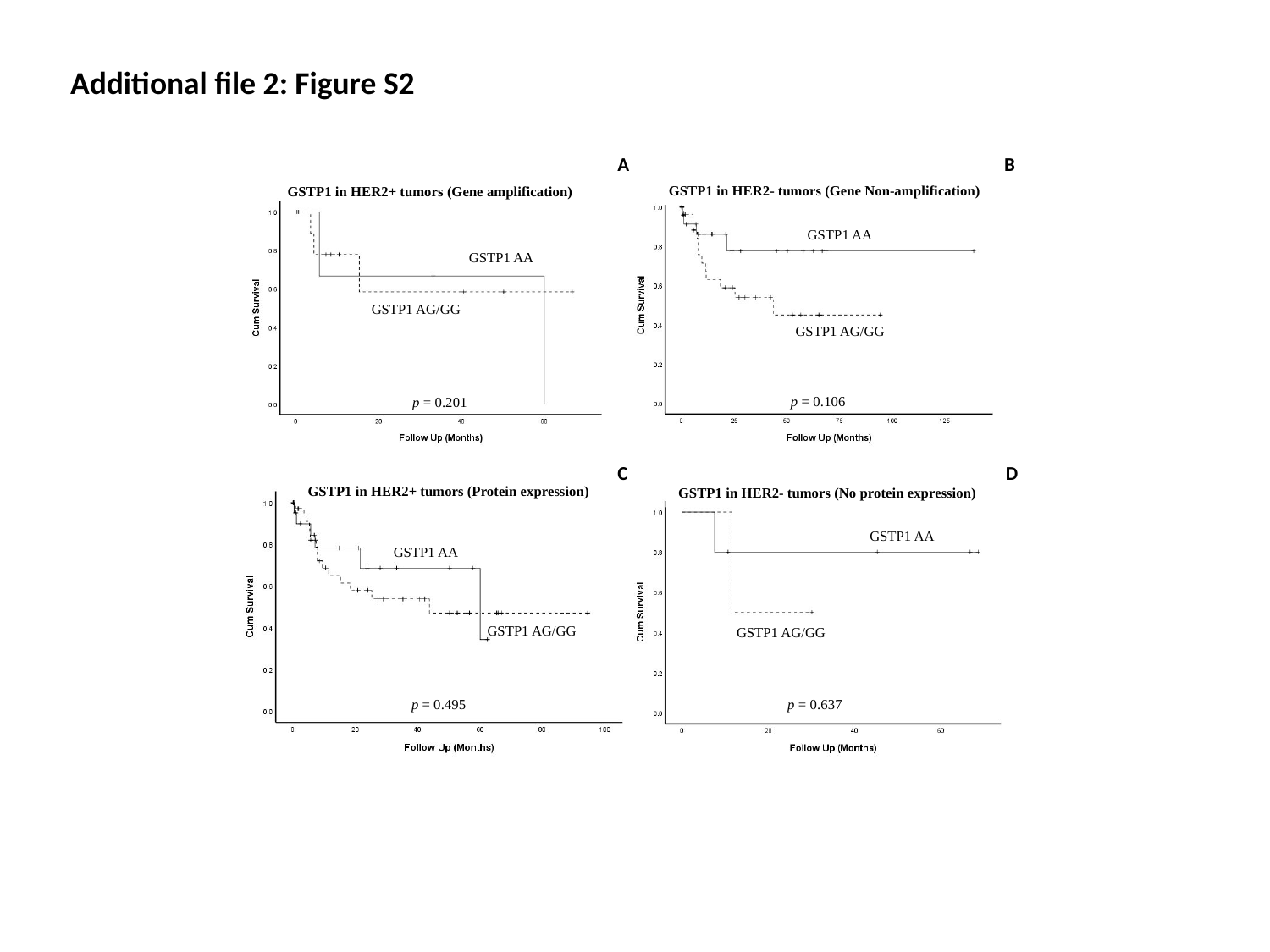

Additional file 2: Figure S2
B
GSTP1 in HER2- tumors (Gene Non-amplification)
GSTP1 AA
GSTP1 AG/GG
p = 0.106
A
GSTP1 in HER2+ tumors (Gene amplification)
GSTP1 AA
GSTP1 AG/GG
p = 0.201
C
GSTP1 in HER2+ tumors (Protein expression)
GSTP1 AA
GSTP1 AG/GG
p = 0.495
D
GSTP1 in HER2- tumors (No protein expression)
GSTP1 AA
GSTP1 AG/GG
p = 0.637
